# Supplementary material for: The Study of Bluetongue Virus (BTV) and Epizootic Hemorrhagic Disease Virus (EHDV) Circulation and Vectors at the Municipal Parks and Zoobotanical Foundation of Belo Horizonte, Minas Gerais, Brazil (FPMZB-BH)
Source: Viruses. 2024 Feb 15;16(2):293. doi: 10.3390/v16020293 (PMC10892844; doi:10.3390/v16020293)
Supplement: Supplementary file 1 [file viruses-16-00293-s001.zip › viruses-2776914-supplementary.pdf]

Supplementary Materials:

Table S1. Data for each *Culicoides spp*. capture

ID	Date	Month	Sunset	Sunrise	maximum temperature (°C)	minimum temperature (°C)	average temperature (°C)	maximum humidity (%)	minimum humidity (%)	lunar phase	Rain on the day	Average atmospheric pressure. (hPa)	Total *Culicoides spp.* collected
Z1	09/12-10/12	Dez	18:28	05:09	28.1	19.6	23.8	87	45	Crescent	N	1014	25
Z2	20/12-21/12	Dez	18:34	05:13	28.9	20.9	24.9	86	53	Convex waning	N	1014	38
Z3	04/01-05/01	Jan	18:39	05:22	32.9	21.5	27.7	99	45	Crescent	Y	1012	14
Z4	19/01-20/01	Jan	18:40	05:32	34.2	23.1	28.7	80	38	Convex waning	N	1019	128
Z5	02/02-03/02	Fev	18:38	05:41	27.4	21.5	24.5	99	69	Crescent	N	1015	52
Z6	24/02-25/02	Fev	18:28	05:51	25.3	21.4	23.4	99	77	waning	N	1018	189
Z7	10/03-11/03	Mar	18:15	05:56	33.9	22.8	28.4	75	20	Quarto Crescent	N	1020	30
Z8	24/03-25/03	Mar	18:03	06:00	29.6	21.9	25.8	81	52	Convex waning	N	1020	37
Z9	06/04-07/04	Abr	17:51	06:04	32.9	24	28.5	76	41	Crescent	N	1018	9
Z10	20/04-21/04	Abr	17:40	06:08	32.1	19	25.1	82	32	Convex waning	N	1018	4
Z11	05/05-06/05	Mai	17:31	06:13	26.6	18.2	22.4	99	72	Crescent	N	1019	10
Z12	18/05-19/05	Mai	17:26	06:18	21.8	8.2	15	74	27	Convex waning	N	1015	0
Z13	01/06-02/06	Jun	17:23	06:24	29.6	17.7	23.7	99	45	Crescent	N	1019	2
Z14	15/06-16/06	Jun	17:24	06:28	25.8	11.3	18.6	99	39	Convex waning	N	1019	1
Z15	28/06-29/06	Jun	17:27	06:31	25.8	10.9	18.4	91	40	new	N	1021	0
Z16	13/07-14/07	Jul	17:32	06:31	29.2	16.3	22.8	83	29	Convex waning	N	1023	2
Z17	27/07-28/07	Jul	17:37	06:27	28.9	12.9	20.9	85	30	waning	N	1022	1
Z18	10/08-11/08	Ago	17:41	06:20	26.3	13.5	19.9	68	43	Crescent	N	1020	0
Z19	25/08-26/08	Ago	17:45	06:09	29.6	18	23.8	73	34	lua minguante	N	1025	3
Z20	05/09-06/09	Set	17:48	05:59	29.6	18	23.8	71	39	Crescent	N	1026	0
Z21	22/09-23/09	Set	17:51	05:44	27.5	20.3	23.9	91	63	waning	Y	1015	0
Z22	06/10-07/10	Out	17:55	05:31	33.9	20.5	27.2	91	41	Crescent	N	1018	9
Z23	19/10-20/10	Out	17:59	05:21	32.5	21.5	27	81	37	minguante	Y	1019	2
Z24	03/11-04/11	Nov	18:05	05:12	21	16.4	18.7	75	65	Crescent	Y	1022	3
Z25	17/11-18/11	Nov	18:14	05:07	32.9	19.9	26.4	73	41	waning	N	1020	9

Table S2. Information on the nearby enclosure of each trap and the amount of captured *Culicoides* spp. in each one.

Trap	Nearby enclosure	Total of captured *Culicoides* spp
A1	camel and llama	198
A2	fallow deer, red deer, marsh deer, and oryx	36
A3	fallow deer, red deer, marsh deer, and oryx	63
A4	tapirs, lowland paca, giant anteaters, and rheas	39
A5	waterbuck and zebra	124
A6	elephants and white rhinoceros	108

Table S3. Information on *Culicoides* spp. species and groups in relation to gender and gonadotropic stage, collected at FPMZB-BH during the period from December 2021 to November 2022.

Culicoides spp. species	Male	Nulliparous	Engorged	Gravid	Parous	Total
Culicoides of the *guttatus* group (Coquillett), 1904	0	2	1	0	2	**5**
Culicoides venezuelensis Ortíz & Mirsa, 1950	0	1	0	2	9	**12**
Culicoides debilipalpis Lutz, 1913	8	0	3	0	6	**17**
Culicoides foxi Ortíz, 1950	1	0	0	0	17	**18**
Culicoides of the *Limai* group	4	8	3	1	7	**23**
Culicoides pusillus Lutz, 1913	7	5	5	1	14	**32**
Culicoides paraensis (Goeldi), 1905	2	4	6	1	22	**35**
Culicoides insignis Lutz, 1913	5	136	42	22	221	**426**
**Total**	**27**	**156**	**60**	**27**	**298**	**568**

Table S4. Information on *Culicoides* spp. species and groups collected at FPMZB-BH during the period from December 2021 to November 2022, in relation to each month of collection.

Culicoides spp. species	Jan	Feb	Mar	Apr	May	Jun	Jul	Aug	Sep	Oct	Nov	Dec	Total
Culicoides of the *guttatus* group (Coquillett), 1904	0	2	0	0	0	0	0	0	0	1	0	2	5
Culicoides venezuelensis Ortíz & Mirsa, 1950	2	2	0	0	0	0	0	2	0	1	0	5	12
Culicoides debilipalpis Lutz, 1913	0	4	0	0	0	0	0	0	0	7	5	1	17
Culicoides foxi Ortíz, 1950	1	10	7	0	0	0	0	0	0	0	0	0	18
Culicoides of the *Limai* group	5	6	3	4	0	0	0	0	0	0	1	4	23
Culicoides pusillus Lutz, 1913	20	6	5	0	0	0	0	0	0	0	0	1	32
Culicoides paraensis (Goeldi), 1905	18	6	3	1	0	0	2	0	0	2	1	2	35
Culicoides insignis Lutz, 1913	96	205	49	9	10	3	1	1	0	0	5	47	426
**Total**	**142**	**241**	**67**	**14**	**10**	**3**	**3**	**3**	**0**	**11**	**12**	**62**	**568**

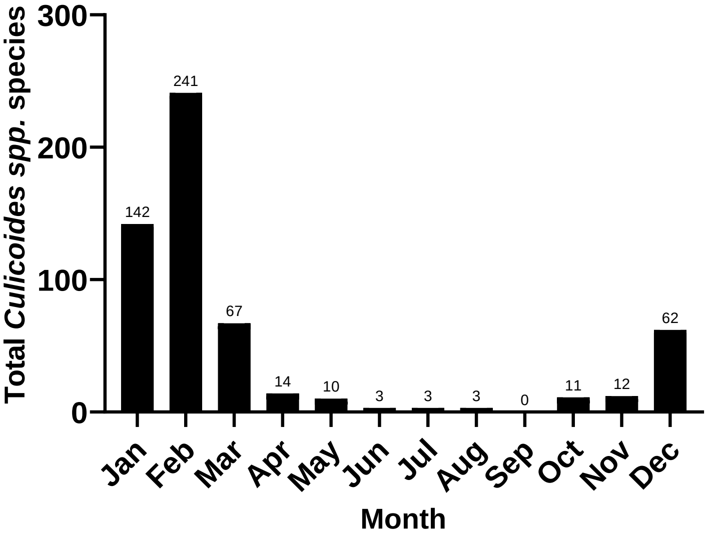

Figure S1. Graph depicting the total number of *Culicoides* spp. collected at FPMZB-BH for each month of the project.

Table S5. Statistical analysis of the variables involving the *Culicoides spp*. captures.

Variable		Univariate analysis		Multivariate analysis	
		RR (95%)	p-Value	RR (95%)	p-Value
**Maximum temperature**		1.01 (0.92 - 1.22)	0.778	-	-
**Minimum temperature**		1.69 (1.47 - 1.94)	0.000*	-	-
**Average temperature**	15 to 20°C	1.26 (1.09 - 1.46)	0.002*	-	-
	25.1 to 30°C	4.5 (0.30 – 66.42)	0.273	-	-
**Temperature ranges**		83.7 (9.30 – 752.96)	0.000*	-	-
**Maximum humidity**		1.06 (1.01 - 1.10)	0.007*	-	-
**Minimum humidity**		1.04 (1.01 - 1.06)	0.001*	-	-
**Medium humidity**		1.04 (1.01 - 1.07)	0.002*	-	-
**lunar phase**		1.77 (0.83 - 3.75)	0.138	-	-
**Lunar luminosity rate**		1.00 (0.99 - 1.02)	0.416	-	-
**Collections on days without rain (Precipitation)**		5.44 (1.89 - 15.66)	0.002*	5.18 (2.00- 13.42)	0.001*
**Traps set outside the enclosure compared to those set inside the enclosures**		2.34 (1.07 - 5.12)	0.034*	-	-
**Water sources near traps**		2.70 (1.03 - 7.08)	0.044*	-	-
**Nearby vegetation**		0.99 (038 - 2.57)	0.979	-	-
**Trap locations ****	1	5.47 (1.59 - 18.85)	0.007*	-	-
	3	1.75 (0.50 - 6.13)	0.381	-	-
	4	1.08 (0.30 - 3.85)	0.902	-	-
	5	3.44 (1.00 - 11.92)	0.051*	-	-
	6	2.86 (0.82 - 9.92)	0.098	-	-
**Presence of accumulation of feces near traps**		3.07 (1.50 - 6.30)	0.002*	2.56 (1.57- 4.18)	0.000*
**Month *****	January	68.51 (15.95 - 294.18)	0.000*	92.65 (22.30 – 384.92)	0.000*
	February	120.50 (28.18- 515.31)	0.000*	116.3 (29.47 – 459.17)	0.000*
	March	33.50 (7.72 - 145.30)	0.000*	31.17 (7.77 – 125.06)	0.000*
	April	6.50 (1.38 - 30.51)	0.018*	6.05 (1.38 – 16.55)	0.017*
	May	5.00 (1.04 - 24.15)	0.045*	5.24 (1.15 - 23.75)	0.032*
	July	1.50 (0.24 - 9.42)	0.665	1.50 (0.25 – 8.98)	0.654
	August	1.50 (0.24 - 9.42)	0.665	1.41 (0.24 – 8.44)	0.703
	September	1.42 (0)	0.982	1.14 (0)	0.992
	October	5.50 (1.15 - 26.27)	0.033*	9.54 (1.99 – 45.55)	0.005*
	November	6.00 (1.27 - 28.39)	0.024*	12.50 (2.61 – 59.79)	0.002*
	December	31.00 (7.14 - 134.67)	0.000*	30.62 (7.61 – 123.23)	0.000*

* Significant results $p < 0.05$.

** Trap 2 was used to compare all traps as it was the one with the lowest number of Culicoides captured, thus being omitted from the table.

*** June was used to compare the months, thus it was omitted from the table.
